# Supplementary material for: Content-rich biological network constructed by mining PubMed abstracts
Source: BMC Bioinformatics. 2004 Oct 8;5:147. doi: 10.1186/1471-2105-5-147 (PMC528731; doi:10.1186/1471-2105-5-147)
Supplement: Additional File 2 — The original results of the above study (non-essential files are deleted to keep the file size under the limit set by BMC bioinformatics). [file 1471-2105-5-147-S2.bz2 › chilibotAdditionalFile2/dip10/168ID7829531E581/html/AKAP5_PRKAR2B.html]

 


 **AKAP5** and **PRKAR2B** 
  
Found 13 abstracts in PubMed, retrieved 10.  
 

 What does Google say? 
 PDF only 
| .edu only 

---

**Interactive relationship** (e.g. stimulation, inhibition, etc)

**Neutral relationship**- The decrease in aromatase mRNA was associated with decreased amounts of mRNA and protein for steroidogenic factor 1 SF 1, a nuclear orphan receptor that binds and trans activates the aromatase promoter, and with the A kinase  [ **AKAP5** ]  subunit type II RII beta  [ **PRKAR2B** ] , which is required for mediating cAMP action in these cells.  Ref: 9029737 Steroids, 1997
- Substitution of Ala for Leu13 or Phe36 generates monomeric RII beta  [ **PRKAR2B** ]  subunits that cannot bind AKAP75  [ **AKAP5** ] .  Ref: 7829531 J Biol Chem, 1995
- Replacement of Val20 Leu21 with Ala Ala produced a dimeric RII beta  [ **PRKAR2B** ]  protein that binds AKAP75  [ **AKAP5** ]  approximately 4% as avidly as wild type RII beta  [ **PRKAR2B** ] .  Ref: 7829531 J Biol Chem, 1995
- The prototypic neuronal anchor protein AKAP75  [ **AKAP5** ]  has a COOH terminal 22 residue RII beta  [ **PRKAR2B** ]  binding tethering site.  Ref: 7829531 J Biol Chem, 1995
- In neurons cAMP dependent protein kinase II beta PKAII beta is sequestered in the dendritic cytoskeleton because the regulatory subunit RII beta  [ **PRKAR2B** ]  of the enzyme is tightly bound by A Kinase  [ **AKAP5** ]  Anchor Proteins AKAPs.  Ref: 7829531 J Biol Chem, 1995
- A kinase  [ **AKAP5** ]  anchor proteins AKAPs have a C terminal binding site for the regulatory subunit RII beta  [ **PRKAR2B** ]  of cAMP dependent protein kinase II beta PKAII beta and an N terminal domain that mediates the targeting and attachment of the anchor protein to intracellular structures.  Ref: 8463292 J Biol Chem, 1993
- A Kinase  [ **AKAP5** ]  Anchor Proteins AKAPs are enriched in forebrain neurons and have distinct high affinity binding domains for the regulatory subunit RII beta  [ **PRKAR2B** ]  of PKAII beta and components of the dendritic cytoskeleton.  Ref: 8509414 J Biol Chem, 1993
- In the human lymphoblastic cell line KE 37, Northern blot analysis with cDNA probes for human regulatory subunits RII alpha RII beta  [ **PRKAR2B** ]  of the cAMP dependent protein kinase A kinase  [ **AKAP5** ]  type II and immunoblotting or immunoprecipitation studies with several antibodies directed against RII alpha and RII beta  [ **PRKAR2B** ]  show that these two isoforms are expressed.  Ref: 8440320 Exp Cell Res, 1993

**Non-interactive relationship** (e.g. studied together, co-existance, homology, etc.)

- Residues containing long aliphatic side chains are essential for the high affinity binding of RII beta  [ **PRKAR2B** ]  by AKAP75  [ **AKAP5** ] .  Ref: 8509414 J Biol Chem, 1993
- Recently characterized A kinase  [ **AKAP5** ]  anchor proteins AKAPs are probable mediators of the sequestration of PKAII beta because they contain a high affinity binding site for the regulatory subunit RII beta  [ **PRKAR2B** ]  of the kinase and a distinct intracellular targeting domain.  Ref: 1333841 Mol Biol Cell, 1992
- A secondary consequence of destabilizing RII beta  [ **PRKAR2B** ]  dimers is the loss of intracellular targeting anchoring capacity because monomers fail to bind AKAP75  [ **AKAP5** ] .  Ref: 7829531 J Biol Chem, 1995
- Mutagenesis, recombinant protein expression, and physicochemical characterization were used to investigate the structural basis for the homodimerization and AKAP75  [ **AKAP5** ]  binding activities of RII beta  [ **PRKAR2B** ] .  Ref: 7829531 J Biol Chem, 1995
- Non transfected cells express a low level of endogenous AKAP79  [ **AKAP5** ] , and approximately 90% of RII alpha and RII beta  [ **PRKAR2B** ]  subunits are isolated in the cell cytosol.  Ref: 8463292 J Biol Chem, 1993
- Certain conservative mutations that should not alter significantly the overall hydrophobicity or helicity of the tethering region e.g. replacement of Leu with Ala diminish the RII beta  [ **PRKAR2B** ]  binding activity of AKAP75  [ **AKAP5** ] .  Ref: 8509414 J Biol Chem, 1993
- We have characterized the targeting anchoring and tethering RII beta  [ **PRKAR2B** ]  binding domains of a prototypic anchor protein AKAP75  [ **AKAP5** ] .  Ref: 8509414 J Biol Chem, 1993
- Other NH2 terminal residues directly modulate the affinity of RII beta  [ **PRKAR2B** ]  dimers for the AKAP75  [ **AKAP5** ]  tethering site.  Ref: 7829531 J Biol Chem, 1995
